# Horizontal and Vertical Distribution of Marine Virioplankton: A Basin Scale Investigation Based on a Global Cruise

**DOI:** 10.1371/journal.pone.0111634

**Published:** 2014-11-03

**Authors:** Yantao Liang, Li Li, Tingwei Luo, Yao Zhang, Rui Zhang, Nianzhi Jiao

**Affiliations:** 1 State Key Laboratory of Marine Environmental Science, Xiamen University, Xiamen, China; 2 Institute of Marine Microbes and Ecospheres, Xiamen University, Xiamen, China; 3 Qingdao Institute of BioEnergy and BioProcess Technology, Chinese Academy of Sciences, Qingdao, China; NERC Centre for Ecology & Hydrology, United Kingdom

## Abstract

Despite the fact that marine viruses have been increasingly studied in the last decade, there is little information on viral abundance and distribution on a global scale. In this study, we report on a global-scale survey covering the Pacific, Atlantic, and Indian Oceans on viral distribution using flow cytometry. Viruses were stained with the SYBR Green I, which targets only dsDNA viruses. The average viral abundance was 1.10±0.73×10^7^ ml^−1^ in global surface oceans and decreased from the areas with high chlorophyll concentration (on average, 1.47±0.78×10^7^ ml^−1^) to the oligotrophic subtropical gyres (on average, 6.34±2.18×10^6^ ml^−1^). On a large-spatial-scale, viruses displayed significant relationships with both heterotrophic and autotrophic picoplankton abundance, suggesting that viral distribution is dependent on their host cell abundance. Our study provided a basin scale pattern of marine viral distributions and their relationship with major host cells, indicating that viruses play a significant role in the global marine ecosystem.

## Introduction

The interests in marine viruses have increased because they are the most abundant group of biological entities in the world’s oceans. The estimated 10^30^ viruses in the ocean contain more carbon than 75 million blue whales [1]. Every second, approximately 10^23^ viral infections occur in the ocean [2]. These infections are a substantial source of mortality in a range of organisms including predominantly autotrophic and heterotrophic microbes. The prokaryotic mortality caused by viral lysis is equal to or sometimes exceeds grazing-induced mortality in marine environments [3], [4]. This process of lytic activities causes the release of dissolved organic carbon (DOC), preventing its flow to higher trophic levels through a “viral shunt” [5], [6]. A previous study indicated that 6–26% of the photosynthetically fixed carbon is “shunted” to the dissolved organic matter (DOM) pool by viral lysis of cells in marine pelagic systems [5]. In addition, the prokaryotic cell components released by viruses might be one of the most important sources of recalcitrant dissolved organic matte (RDOM) in the microbial carbon pump (MCP), which is a conceptual framework of long-term carbon storage in the global ocean [7]. In general, viruses modulate the transfer and transformation of carbon and nutrients in marine microbial food webs and play an important role in biogeochemical cycles and energy flow in the ocean and directly or indirectly affect the global ecosystem.

Over the past two decades, viral ecology has been studied in a wide range of marine habitats including estuary, coastal sea, open sea and extreme environments such as deep oceans [3]. These studies indicate that viruses are truly ubiquitous and their impact on the microbial community can be variable. However, our understanding of virioplankton in the global ocean is still limited, partially due to methodological inconsistency. Currently, there are three major techniques for the numeration of viral particles in environmental samples: transmission electron microscopy (TEM), epifluorescence microscopy (EFM) and flow cytometry (FCM) [3], [8]. Although the data sets obtained by different methods may be positively correlated, they are incomparable with each other. It has been reported that EFM produces significantly higher viral numbers than TEM [9], [10]. Even using the same technique, different protocols cause significant effects on the accuracy of virus enumeration [8], [11]. Thus, the methodological discrepancy among different studies might to be one of the major difficulties for estimating marine viral abundance and determining the ecological implications on the global scale. In addition, there are still arguments regarding the factors controlling viral distribution and which factor plays a more important role on the large spatial scale [12]–[16]. Moreover, reports of vertical variation of viral abundance in the whole water column, especially in the deep sea, are few [13], [15], [17]–[21]. In this study, we performed a systematic survey of the abundances of viruses and their major host cells (picoplankton = heterotrophic prokaryotes + *Prochlorococcus* + *Synechococcus* + picoeukaryotes, Fig. S1) using flow cytometry during a global expedition cruise covering various coastal and oceanic environments. The correlations between viral abundance and host cell abundance or environmental factors were also investigated.

## Materials and Methods

### Study area and sampling

The samples were collected from a global expedition (December 2010–December 2011) aboard *R/V Ocean No. 1* (Fig. S2). The South China Sea samples were taken during December 2010, the Indian Ocean samples were taken from December 2010 to January 2011, the Atlantic Ocean samples were taken from March to June 2011 and the Pacific samples were taken from July to December 2011. The samples were collected from the vertical profile at 7 to 23 depths from epipelagic (0–200 m), mesopelagic (200–1000 m), and bathypelagic (1000–3150 m) zones at 22 stations including 4 stations in the Indian ocean, 13 stations at the south Atlantic gyre and 5 stations at the eastern equatorial Pacific upwelling area (No specific permissions were required for these locations/activities). The samples (1.5 ml) for flow cytometry analysis were fixed with glutaraldehyde (final concentration: 0.5%), flash-frozen in liquid nitrogen and stored at −80°C prior to analysis [8].

### Viral and host cell counting using flow cytometry

Viruses, heterotrophic prokaryotes and autotrophic picoplankton were analyzed on an Epics Altra II flow cytometer (Beckman Coulter, USA) with a 306C-5 argon laser (Coherent, USA). Virus enumeration was performed according to the method of Brussaard [8]. Once thawed at 37°C, the samples were diluted 5 to 50 times in 0.02-µm filtered Tris-EDTA buffer (pH = 8, Sigma-Aldrich). After staining with the SYBR Green I (1/20000 final concentration, Molecular Probes, which targets only dsDNA viruses), the diluted samples were heated for 10 min in the dark at 80°C and then cooled for 5 min prior to analysis. The samples were run at a flow rate of 0.1–1 ml h^−1^ (using HARVARD PHD2000 APPARATUS). For the enumeration of viruses, we set the discriminator to green fluorescence and set all parameters on logarithmic amplification. The typical settings on an Epics Altra II flow cytometer are forward scatter (PMT4, for the accurate detection of the weak signal of forward scatter of picoplankton, we changed the PMT4 as the forward scatter detector) = 400, side scatter (PMT1) = 590, green fluorescence (PMT2) = 720, orange fluorescence (PMT3) = 500, and red fluorescence (PMT5) = 910. The viruses were discriminated on the basis of the green fluorescence and side scatter signal. Usually, two subgroups of viruses were observed in the cytograms (Fig. S3A–C). The group with higher fluorescence was called the HFV group, and the group with lower fluorescence was called the LFV group; total viruses = HFV + LFV.

Heterotrophic prokaryote enumeration was performed according to the method of Marie et al. [22]. The samples were stained with the SYBR Green I (1/10000 final concentration, Molecular Probes) for 15 min in the dark at room temperature prior to analysis. The samples were run at a flow rate of 0.1–1 ml h^−1^. For the enumeration of heterotrophic prokaryotes, we set the discriminator to green fluorescence and set all parameters on logarithmic amplification. The typical settings on an Epics Altra II flow cytometer are PMT4 = 430, PMT1 = 400, PMT2 = 550, PMT3 = 640, and PMT5 = 1000. Heterotrophic prokaryotes were identified in plots of red fluorescence vs. green fluorescence (Fig. S3D–E). The autotrophic picoplankton abundance was determined according to the method of Jiao et al. [23]. We set the discriminator to red fluorescence and all parameters on logarithmic amplification. The typical settings on an Epics Altra II flow cytometer are PMT4 = 400, PMT1 = 350, PMT2 = 570, PMT3 = 750, and PMT5 = 1000. Autotrophic picoplankton are identified in plots of side scatter vs. red fluorescence and orange fluorescence vs. red fluorescence (Fig. S3F–G). Fluorescent microspheres (Molecular Probes Inc.) with a diameter of 1 µm were added to all samples as an internal standard. The data were analyzed with EXPOTM32 MultiCOMP software (Beckman Coulter, USA).

The ratios of a different viral group and their possible corresponding host cell abundance were calculated, including the ratio of HFV and picoeukaryotic abundance (HVEukR), the ratio of LFV and prokaryotes abundance (prokaryotes abundance = heterotrophic prokaryotes + *Prochlorococcus* + *Synechococcus* abundance, LVProkR) and the ratio of total viruses and total picoplankton abundance (total picoplankton abundance = picoeukaryotes + prokaryotes abundance, VPR).

### Statistical analysis

An analysis of variance (ANOVA) and independent *t*-test were employed to compare the differences in parameters using the SPSS (18.0) software package (SPSS Inc., Chicago, IL, USA). A Pearson correlation analysis was applied to assess the degree of correlation among the investigated parameters. A linear regression analysis for log-transformed viral abundance and log-transformed host cell abundance was performed using Sigma-Plot 10.0. The distance-based multivariate analysis for a linear model using forward selection (DISTLM forward) was applied to test the relationships between viral abundance and biotic and abiotic environmental parameters [24].

## Results and Discussion

### Horizontal distribution pattern of viral abundance and its association with host cells

**Figure 1:**
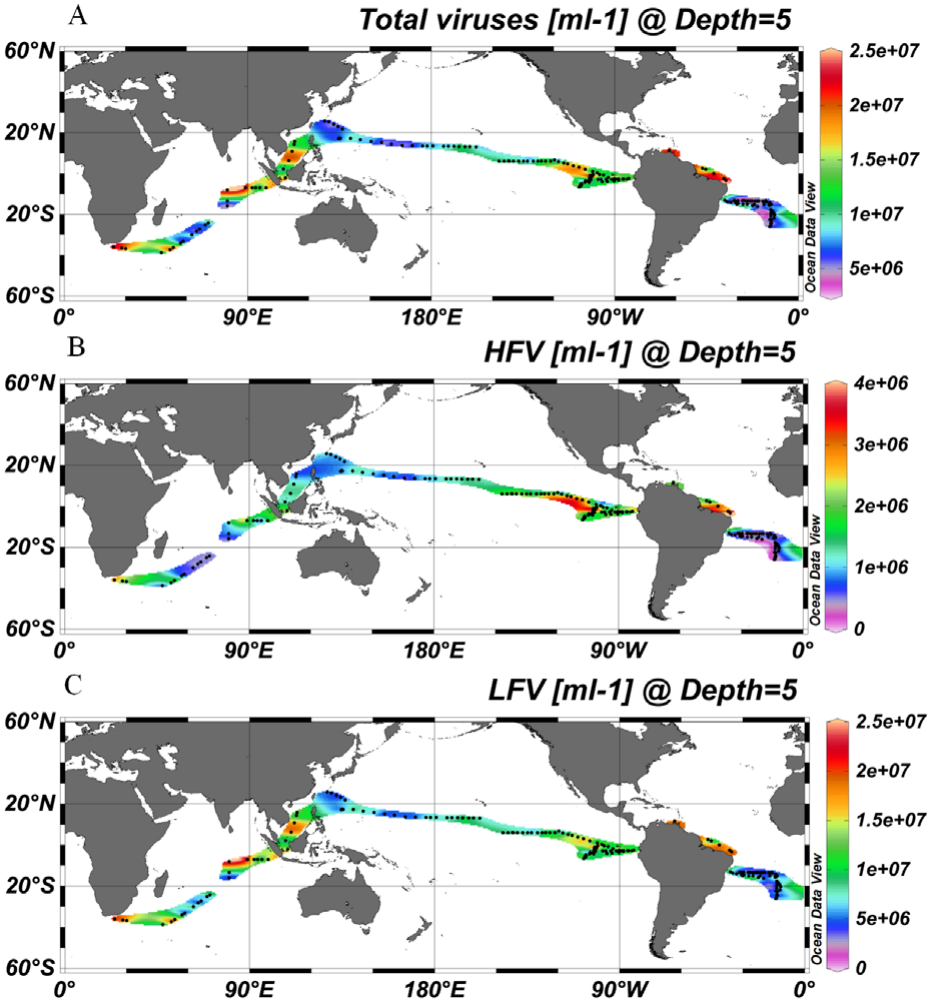
A global distribution pattern of the total viral abundance (A), HFV abundance (B) and LFV abundance (C). The map was generated with Ocean Data View software [40]. Abbreviations: HFV, high fluorescence viruses; LFV, low fluorescence viruses.

**Figure 2:**
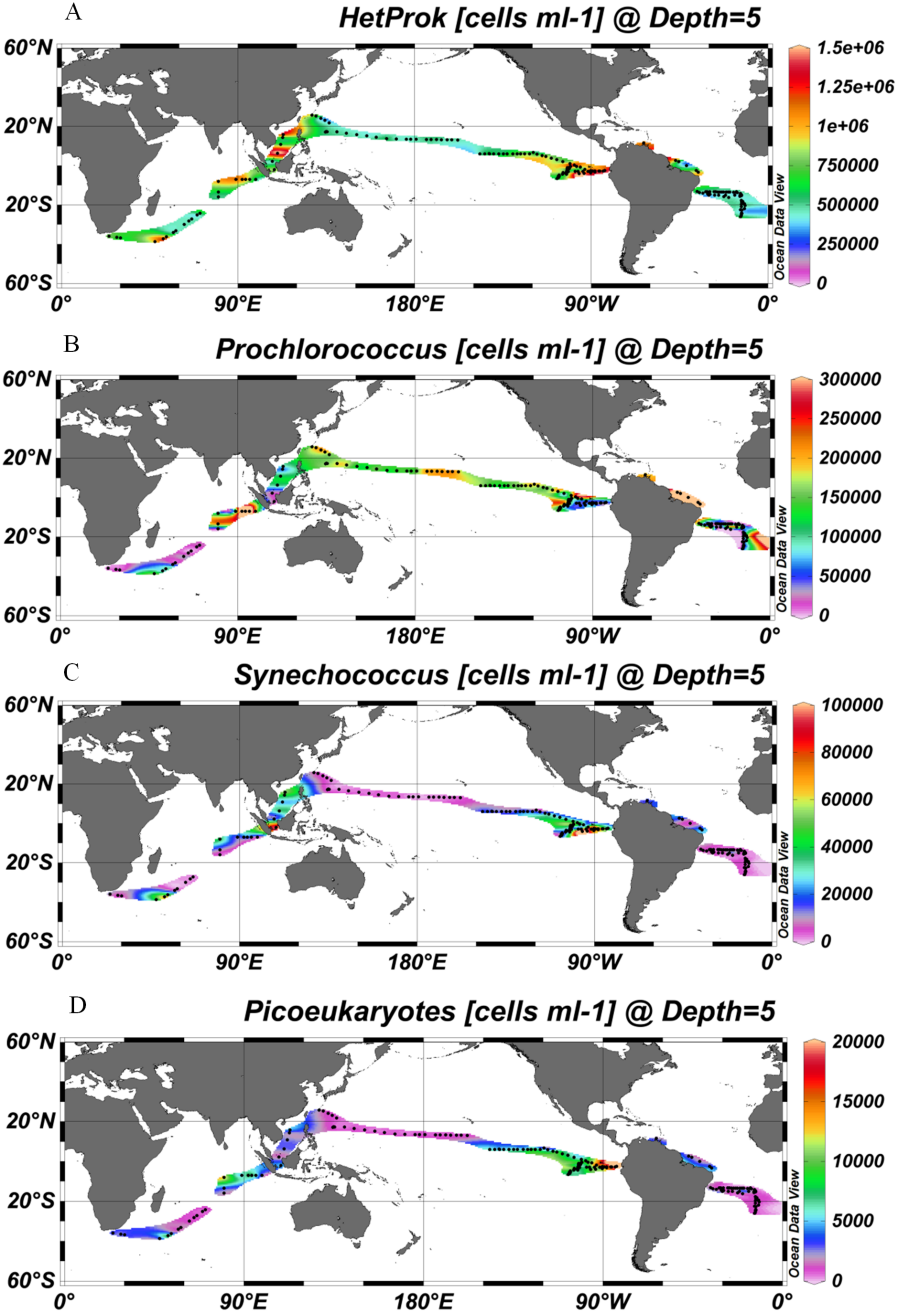
A global distribution pattern of the heterotrophic prokaryotic cell abundance (A), *Prochlorococcus* (B), *Synechococcus* (C) and picoeukaryotes abundance (D). The map was generated with Ocean Data View software [40].

A total of 206 samples from surface water of the global ocean and 252 discrete samples from 22 depth profiles were collected and analyzed by FCM. Viruses were stained with the SYBR Green I. Although SYBR Green I targets only dsDNA viruses [8] and SYBR Gold would also be able to detect ssDNA and RNA viruses [25], Brussaard conducted a detailed evaluation of the two sensitive nucleic acid stains and recommended SYBR Green I for viral analysis [8]. Currently, SYBR Green I is widely used for viral enumeration [13]–[15],. To obtain comparable results to those of other studies, we stained the viral samples with SYBR Green I. Generally, the viral abundance of surface water varied from 2.54×10^6^ to 7.15×10^7^ ml^−1^ and was higher (1.47±0.78×10^7^ ml^−1^) in the coastal/shelf waters and the presumed ocean upwelling waters with higher surface chlorophyll concentration (>0.07 mg m^−3^) [27] than (6.34±2.16×10^6^ ml^−1^) in the subtropical oceanic gyres (surface chlorophyll concentration ≤0.07 mg m^−3^, P<0.001, independent *t*-test, Table 1, Fig. 1A) [28]. The distribution patterns of host cells (e.g., heterotrophic prokaryotes, *Synechococcus* and picoeukaryotes) were similar to that of the total viral abundance (Pearson correlation, P<0.001, Fig. 1A, 2A, and 2C–D).

**Table 1:** Mean values (± standard deviations) of microbial parameters in the surface waters of the study areas.

Provinces	Parameters^a^	Proc*	Syn*	Euk*	HetProk*	Prokaryotes*	Picoplankton*	HFV*	LFV*	Total viruses*	HTP %*	HVEukR*	LVProkR*	VPR*
Coastal/shelf/upwelling	Abundance	1.32±0.90×10^5^	3.44±3.52×10^4^	7.50±4.70×10^3^	8.73±3.96×10^5^	1.02±0.36×10^6^	1.02±0.36×10^6^	2.08±0.92×10^6^	1.26±0.72×10^7^	1.47±0.78×10^7^	15.01±4.70	372±279	12.84±5.80	14.93±6.46
Gyres	Abundance	9.26±7.74×10^4^	3.46±2.72×10^3^	1.00±0.70×10^3^	4.59±0.90×10^5^	5.55±1.23×10^5^	5.56±1.23×10^5^	6.25±3.29×10^5^	5.71±1.99×10^6^	6.34±2.18×10^6^	9.78±3.63	793±597	10.59±3.99	11.68±4.21

a Unit of abundance: cells ml^−1^. Abbreviations: Proc, *Prochlorococcus*; Syn, *Synechococcus*; Euk, picoeukaryotes; HetProk, heterotrophic prokaryotes; HFV, higher fluorescence virus; LFV, low fluorescence virus; HTP, the percent of HFV to total viruses; HVEukR, the ratio of HFV and picoeukaryotes abundance; LVProkR, the ratio of LFV and prokaryotic abundance; VPR, the ratio of total viruses and picoplankton abundance.

*indicated the parameters for Coastal/shelf/upwelling and gyres are statistically different (independent *t* test, P<0.01).

Based on FCM analysis, the low DNA-content LFV group accounted for approximately 90% of the total viruses in the surface waters and averaged 1.26±0.72×10^7^ and 5.71±1.99×10^6^ ml^−1^ in the coastal/shelf/upwelling (CSU) areas and the subtropical gyres, respectively (P<0.001, independent *t* test, Table 1, Fig. 1B). The high DNA-content HFV group is usually considered to comprise viruses of autotrophic eukaryotes because of the higher genome sizes and consistent positions in the FCM biplots with algal viruses [29], . In our investigation, the CSU waters had a higher HFV abundance and percent of total viruses (2.08±0.92×10^6^ ml^−1^ and 15.01±4.70%) than the gyre waters (6.25±3.29×10^5^ ml^−1^ and 9.78±3.63%, P<0.001, independent *t*-test, Table 1, Fig. 1C).

The absolute values of latitude were negatively correlated with the abundance of HFV, total viruses, *Prochlorococcus*, *Synechococcus*, picoeukryotes, and heterotrophic prokaryotes (Pearson correlation, *r* = −0.489, −0.189, −0.216, −0.342, −0.555 and −0.291, P<0.01, respectively), which is similar with the results that have been reported in the north Atlantic Ocean [13]. They found that the latitude contributed to the variation of total viral abundance in the epipelagic layer, although the contribution of latitude was low (*r^2^* = 0.05; P = 0.04).

**Figure 3:**
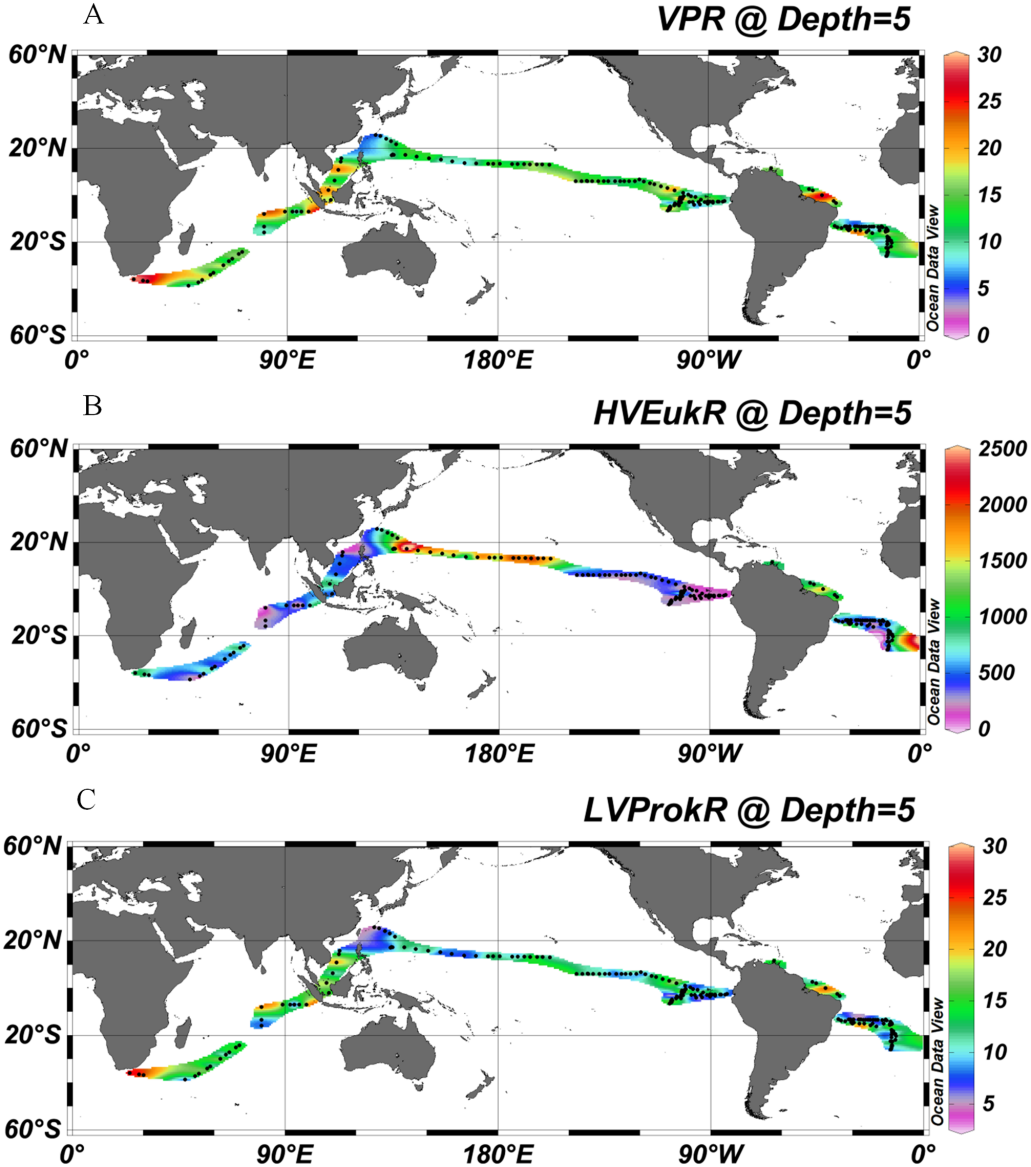
A global distribution pattern of the VPR (A), HVEukR (B) and LVProkR (C). The map was generated with Ocean Data View software [40]. Abbreviations: VPR, the ratio of total viral abundance and picoplankton abundance (heterotrophic prokaryotes + *Prochlorococcus* + *Synechococcus* + picoeukaryotes); HVEukR, the ratio of high fluorescence viral abundance and picoeukaryotes abundance; LVProkR, the ratio of low fluorescence viral abundance and prokaryotic abundance (heterotrophic prokaryotes + *Prochlorococcus* + *Synechococcus*).

The ratio of total viruses and total picoplankton abundance (VPR) varied from 4.95 to 34.94 in the study areas and showed the same pattern as viral abundance. The CSU waters had higher VPR (14.93±6.46) than the subtropical gyres (11.68±4.21) (P<0.001, independent *t*-test, Table 1, Fig. 3A), which is in agreement with the studies in the Adriatic Sea and the Southern Ocean [16], [31]. LVProkR was also higher in the CSU waters (12.84±5.80) than the subtropical gyres (10.59±3.99) (P<0.001, independent *t*-test, Table 1, Fig. 3B). These results suggest that hosts sustain greater numbers of viruses under the environments favoring fast growth and high productivity. It has been reported that the increases in viral production might be due, in part, to a higher burst size in the high-nutrient waters [3]. Meanwhile, high autotrophic host abundance may induce a higher concentration of algal viruses and cyanophages, which could also result in a higher VPR [32]. In our study, the observed low VPR in the oligotrophic gyres agreed with the results reported in the low-nutrient marine environment [9], [33]. The reasons might include the low contact rates between viruses and prokaryotes because of the low abundance of host cells, the presumably non-lytic life strategies of viruses (including chronic infections, lysogeny and pseudolysogeny), and the lower frequency of prokaryotes with mature phage in such environments [34]. HVEukR was higher than the VPR and LVProkR and varied from 127 to 3084 in the surface waters (P<0.001, independent t-test), which is similar to the reported burst sizes of eukaryotic viruses (ranged from 72–100,000 cell^−1^, P<0.001, one-way ANOVA) [29], [30]. The distribution pattern of HVEukR was different from the VPR and higher in the subtropical gyres (793±597) than in the CSU waters (372±279) (P<0.001, independent t-test, Table 1, Fig. 3A–C).

**Figure 4:**
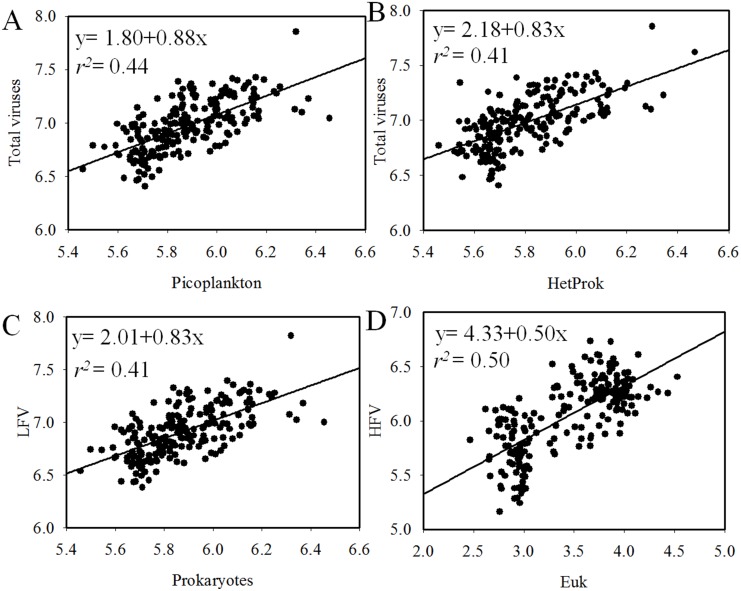
Relation between the abundance of total viruses and picoplankton (A), total viruses and HetProk (B), LFV and prokaryotes (C) and HFV and Euk (D) in surface waters. The fitting function used is: y = ax+b, all the data were log transformed. Abbreviations: HetProk, heterotrophic prokaryotes; LFV, low fluorescence viruses; HFV, high fluorescence viruses; Euk, picoeukaryotes.

Over the entire sampling area, viral abundance displayed significant correlation with heterotrophic prokaryotic abundance (linear regression *r* ^2^ = 0.41, Pearson correlation, P*<*0.001, Fig. 4B), which is consistent with most previous studies [16], [35], [36] and suggests a tight coupling between heterotrophic prokaryotic and viral concentrations. The total picoplankton abundance (heterotrophic prokaryotes + *Prochlorococcus* + *Synechococcus* + picoeukayotes) has a slightly better explanation of total viral abundance than heterotrophic prokaryotic abundance (linear regression *r* ^2^ = 0.44, Pearson correlation, P<0.001, Fig. 4A–B). This indicated that viral distribution was dependent on both heterotrophic and autotrophic host cell abundance in the surface ocean. These observations were in agreement with the results of the central Pacific transects and the northwestern Sargasso Sea, which determined that autotrophic host cell abundance might contribute significantly to the variation in viral abundance [12], [14]. The multivariate multiple regression analysis (DISTLM-*forward*) indicated that heterotrophic prokaryotic and *Prochlocococcus* abundance together explained 36% of the variability in viral abundance in the surface waters of the global ocean (Table 2 & S1). This further suggested that viral distribution was dependent on both heterotrophic and autotrophic host cell abundance in the surface ocean.

**Table 2:** Results of the multivariate multiple regression analysis with forward selection (DISTLM forward) to explain the variability in viral abundance in the surface waters of coastal/shelf/upwelling and oceanic gyre water.

*Provinces*	*Selected variables*	*Pseudo-F*	P	r^2^	*Cumulative*
Global ocean (n = 205)	HetProk	110.1725	0.0001	0.3518	0.3518
	Proc	4.7114	0.0279	0.0148	0.3666
Coastal/shelf/Upwelling(n = 115)	HetProk	16.6441	0.0010	0.1284	0.1284
	Euk	7.9945	0.0140	0.0581	0.1865
Gyres (n = 90)	Proc	12.5465	0.0030	0.1248	0.1248
	Syn	21.5631	0.0010	0.1738	0.2986

Abbreviations: Proc, *Prochlorococcus*; Syn, *Synechococcus*; Euk, picoeukaryotes; HetProk, heterotrophic prokaryotes.

The response variable was log-transformed and the resulting data were converted into Euclidian distance similarities matrices. The Pseudo-F and the *P*-values were obtained by permutation (n = 999).

### Vertical distribution pattern of viral abundance and its association with host cell abundance

**Figure 5:**
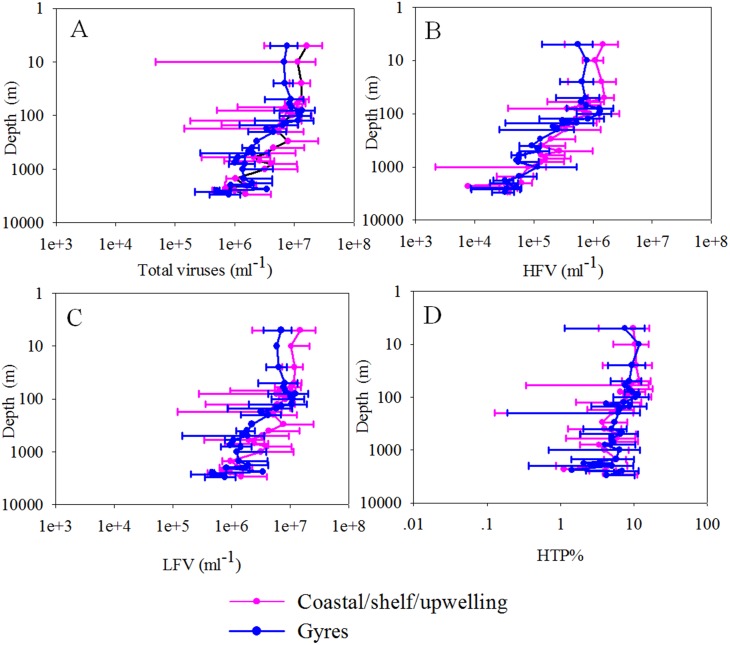
Depth profiles of total viral abundance (A), HFV abundance (B), LFV abundance (C) and HTP% (D) in the whole water column (0–3000 m) of the 22 vertical-sampling stations, respectively. The solid line is the average value, while the error bars represent 95% confidential intervals. The water depths of samples were considered as 2500 m when the bottom depths were between 2500 and 2700 m and as 3000 m when the depths were between 2900 and 3150 m. Abbreviations: HFV, high fluorescence viruses; LFV, low fluorescence viruses. HTP%, the percent of HFV to total viruses.

**Figure 6:**
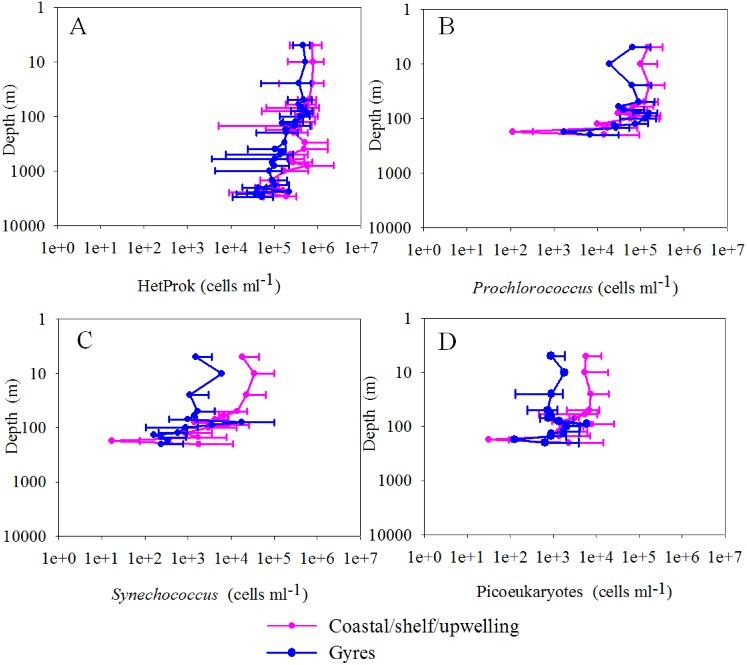
Depth profiles of heterotrophic prokaryotic abundance (A), *Prochlorococcus* abundance (B) *Synechococcus* abundance (C) and picoeukaryotes abundance (D) in the whole water column (0–3000 m) of the 22 vertical-sampling stations, respectively. The solid line is the average value, while the error bars represent 95% confidential intervals. The water depths of samples were considered as 2500 m when the bottom depths were between 2500 and 2700 m and as 3000 m when the depths were between 2900 and 3150 m.

**Figure 8:**
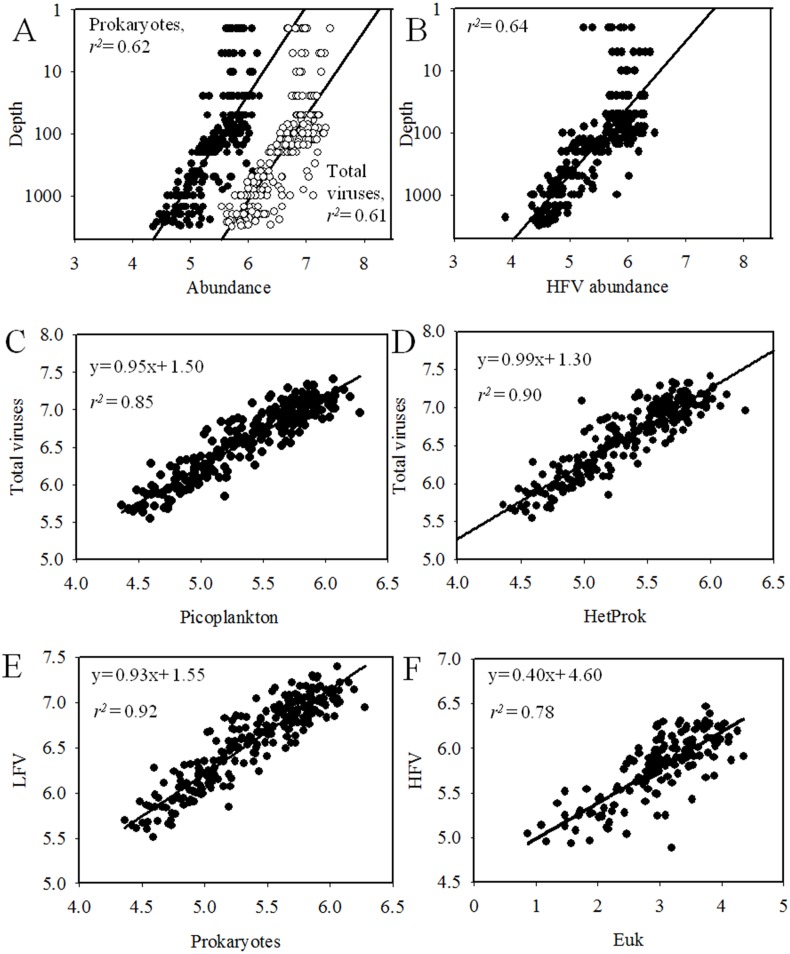
Relation between the abundance of prokaryotes and total viruses versus depth (A), LFV versus depth (B), total viruses versus picoplankton (C), total viruses versus HetProk (D), LFV and prokaryotes (E) and HFV abundance and Euk (F) in the whole water column of 22 vertical-sampling stations. The fitting function used is: y = ax+b, all the data were log transformed. Abbreviations: LFV, low fluorescence viruses; HFV, high fluorescence viruses; HetProk, heterotrophic prokaryotes; Euk, picoeukaryotes.

The abundance of viruses significantly decreased with depth from an average of 8.94±4.69×10^6^ ml^−1^ in the epipelagic waters to 1.11±0.78×10^6^ ml^−1^ in the bathypelagic waters (ANOVA on rank, P<0.001, Fig. 5A), which is comparable to the abundance reported in the Atlantic and Pacific Oceans and the northwestern Mediterranean Sea [13], [15], [17]–[19], [21], [37]. The abundance of high-fluorescence viruses significantly decreased with depth (linear regression *r* ^2^ = 0.64, Pearson correlation, P<0.001, Fig. 5B and 8B) and the percentage of high-fluorescence viruses decreased from an average of 8.34±3.21% in the epipelagic layer to 4.80±2.03% (ANOVA on rank, P<0.001, Fig. 5D) in the bathypelagic layer, which is in agreement with previous studies from the Atlantic Ocean [13], [15]. The LFV abundance decreased with depth from an average of 8.25±4.12×10^6^ ml^−1^ in the epipelagic waters to 1.07±0.77×10^6^ ml^−1^ in the bathypelagic waters (ANOVA on rank, P<0.001, Fig. 5C). Comparatively, the prokaryotic abundance decreased by more than an order of magnitude in the vertical profiles (ANOVA on rank, P<0.001, Fig. 6A).

**Figure 7:**
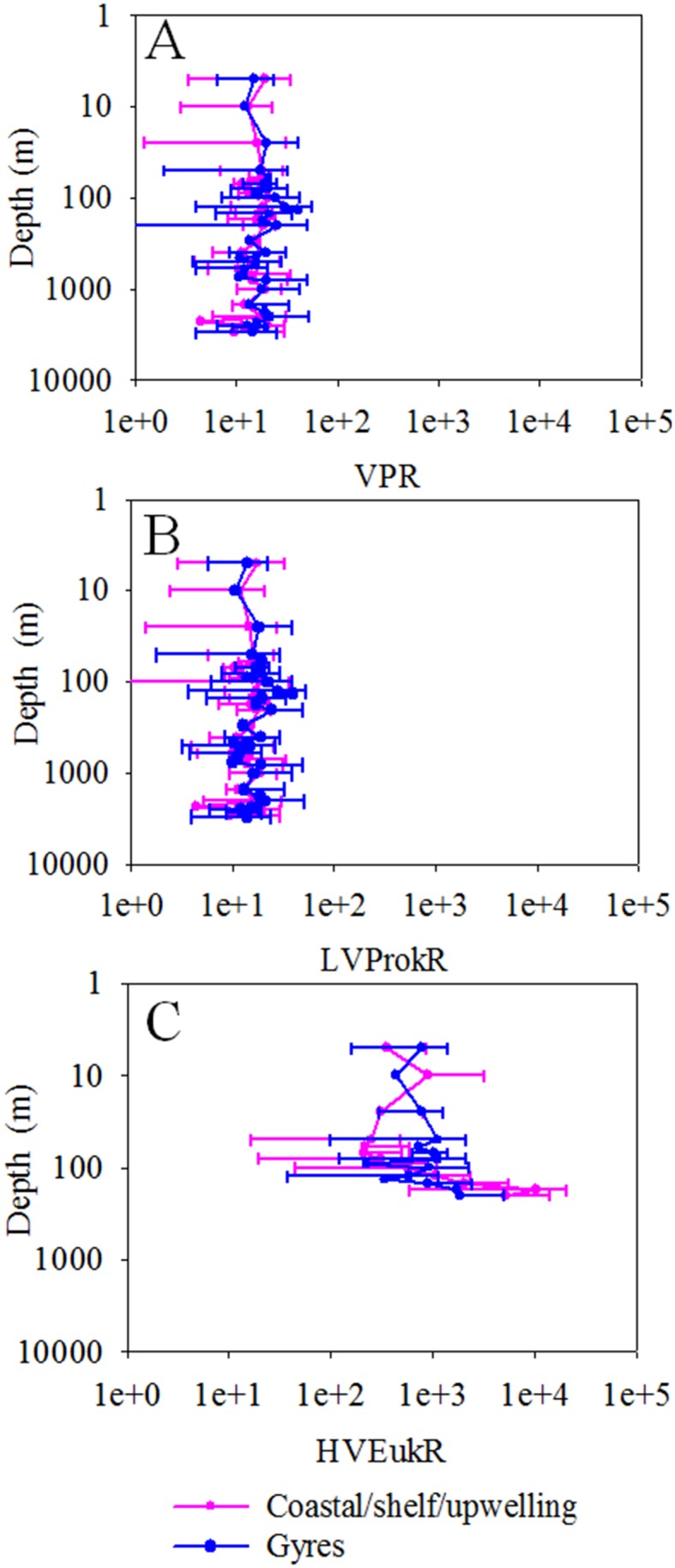
Depth profiles of VPR (A), LVProkR (B) and HVEukR (C) in the whole water column (0–3000 m) of the 22 vertical-sampling stations, respectively. The solid line is the average value, while the error bars represent 95% confidential intervals. The water depths of samples were considered as 2500 m when the bottom depths were between 2500 and 2700 m and as 3000 m when the depths were between 2900 and 3150 m. Abbreviations: VPR, the ratio of total viral abundance and picoplankton abundance (heterotrophic prokaryotes + *Prochlorococcus* + *Synechococcus* + picoeukaryotes); HVEukR, the ratio of high fluorescence viral abundance and picoeukaryotes abundance; LVProkR, the ratio of low fluorescence viral abundance and prokaryotic abundance (heterotrophic prokaryotes + *Prochlorococcus* + *Synechococcus*).

The VPR slightly decreased with depth from an average of 19.0±8.2 over all the stations in the epipelagic layer to 16.2±7.9 in the bathypelagic layer (ANOVA on rank, P<0.05, Fig. 7A). The highest VPR was usually displayed between 70 and 200 m in the Atlantic oceanic gyre water (average: 25.3±9.9, range: 12.4–47.6, Fig. 7A). The vertical distribution pattern of LVProkR is similar to that of VPR and slightly decreased with depth from an average of 17.5±7.7 over all the stations in the epipelagic layer to 15.5±7.8 in the bathypelagic layer (ANOVA on rank, P>0.05, Fig. 7A–B). These results are in agreement with the results reported in the northwestern Mediterranean Sea, the Arctic and the western Pacific [18], [37]–[39]. Similar and slightly decreasing or increasing trends of VPR from the epipelagic layer to the deep sea waters were observed in the eastern and northern basin of the Atlantic [13], [15], [19], central Pacific [17] and the Mediterranean Sea [18], [20], [26]. Furthermore, VIEukR was relatively stable in the epipelagic zone of gyre waters (980±574) and increased with depth in the relatively eutrophic CSU waters (Fig. 7C) due to the different distribution of picoeukaryotes in these two environments (Fig. 6D).

Viral abundance was negatively related to depth (linear regression *r* ^2^ = 0.61, Pearson correlation, P<0.001, Fig. 8A) and positively related to heterotrophic prokaryotic abundance (linear regression *r* ^2^ = 0.81, Pearson correlation, P<0.001, Fig. 8D). In the epipelagic waters, the highest viral abundance usually displayed between 50 and 100 m in the oceanic gyre waters and above 50 m in the highly primary productive upwelling waters (Fig. 5A). This roughly matched the depth of the subsurface chlorophyll maxima (corresponding to the peak value of *Prochlorococcus*, *Synechococcus* and picoeukaryotes abundance) observed in our study (Fig. 6B–D). The subsurface peaks in virioplankton abundance around the depth of the chlorophyll maxima were observed previously [12], [35], [37]. This finding could be due to the relatively high virus loss due to sunlight in the surface area and a high viral production rate because of the peak of host cell abundance and productivity at chlorophyll maxima layers. The results of the correlation analysis and DISTLM-*forward* analysis also showed that a vertical variation of viral abundance in the epipelagic waters was dependent on both heterotrophic and autotrophic host cell abundance. Significant positive correlations were found between the viral abundance and the cell concentration of *Prochlorococcus*, *Synechococcus*, picoeukaryotes and heterotrophic prokaryotes (linear regression *r* ^2^ = 0.44, *r* ^2^ = 0.37, *r* ^2^ = 0.55, and *r* ^2^ = 0.46, respectively; Pearson correlation, P<0.001). DISTLM-*forward* analysis showed that the variables explaining most of the variability (57%) in viral abundance in the epipelagic zone were the abundance of heterotrophic prokaryotes, *Prochlorococcus*, *Synechococcus* and picoeukaryotes and depth (Table 3 & S1). In the meso- and bathypelagic waters, viral abundance was significantly and positively correlated with heterotrophic prokaryotic abundance (linear regression *r* ^2^ = 0.73, Pearson correlation, P<0.001). DISTLM-*forward* analysis showed that heterotrophic prokaryotic abundance explained 54% and 30% of the variability in viral abundance in the meso- and bathypelagic waters, respectively, and that depth contributed to another 3% of the variation in viral abundance in the mesopelagic waters (Table 3 & S1).

**Table 3:** Results of the multivariate multiple regression analysis with forward selection (DISTLM forward) to explain the variability in viral abundance throughout the water column (total) and in specific depth layers in different geographic regions.

*Provinces*	*Selected variables*	*Pseudo-F*	P	r^2^	*Cumulative*
Global ocean (n = 252)	Depth	381.1793	0.0010	0.6039	0.6039
	HetProk	172.8139	0.0010	0.1623	0.7662
	Proc	33.3812	0.0010	0.0277	0.7939
	Euk	5.2376	0.0220	0.0043	0.7982
	Syn	7.5410	0.0060	0.0060	0.8042
Epi (n = 152)	HetProk	106.2202	0.0010	0.4146	0.4146
	Proc	28.6149	0.0010	0.0943	0.5089
	Euk	8.5936	0.0040	0.0270	0.5358
	Depth	7.4071	0.0070	0.0223	0.5581
	Syn	4.1951	0.0410	0.0123	0.5704
Meso (n = 56)	HetProk	62.2510	0.0010	0.5355	0.5355
	Depth	4.1708	0.0460	0.0339	0.5694
Bathy (n = 44)	HetProk	17.9534	0.0010	0.2995	0.2995
Coastal/shelf/upwelling (n = 120)	HetProk	168.3646	0.0010	0.5879	0.5879
	Depth	100.0706	0.0010	0.1900	0.7779
	Euk	24.4152	0.0010	0.0386	0.8165
	Syn	3.2953	0.0700	0.0051	0.8216
	Proc	5.1984	0.0290	0.0078	0.8294
Epi (n = 74)	HetProk	80.4848	0.0010	0.5278	0.5278
	Depth	20.4735	0.0010	0.1057	0.6335
	Euk	14.3137	0.0010	0.0622	0.6957
	Syn	11.9527	0.0010	0.0449	0.7406
Meso (n = 28)	HetProk	43.7601	0.0010	0.6273	0.6273
Gyres (n = 132)	HetProk	245.4824	0.0010	0.6538	0.6538
	Depth	76.5997	0.0010	0.1290	0.7828
	Proc	20.5376	0.0010	0.0300	0.8128
Epi (n = 78)	Proc	84.3087	0.0010	0.5259	0.5259
	Euk	5.7289	0.0120	0.0336	0.5596
	Depth	3.9734	0.0420	0.0224	0.5820
Meso (n = 28)	Depth	9.5536	0.0020	0.2687	0.2687
Bathy (n = 26)	HetProk	40.7493	0.0010	0.6293	0.6293

Abbreviations: Proc, *Prochlorococcus*; Syn, *Synechococcus*; Euk, picoeukaryotes; HetProk, heterotrophic prokaryotes.

The response variable was log-transformed and the resulting data were converted into Euclidian distance similarities matrices. The Pseudo-F and the *P*-values were obtained by permutation (n = 999).

Altogether, our study suggested that the horizontal and vertical distributions of virioplankton at large spatial scales are controlled by dynamic host-virus relationships. Virioplankton play an active and important role in marine ecosystems and biogeochemical cycling in all of the global ocean, including meso- and bathypelagic oceans. Correlative analyses of the abundances of total viruses and their potential host cells suggest possible controls on marine viral dynamics. Further phylogenetic quantification of phages, experimental determination of viral pressure on lineages of each host and additional modeling exercises using these data are required to resolve the drivers of the spatial variability in total viruses and their ecological effects on the biogeochemical cycles in the global ocean.

## Supporting Information

Figure S1 **The hierarchical tree of how the samples fractionated.**
(TIF)

Figure S2 **Sampling stations of the global cruise.** The blue points represent the stations where surface samples were collected. The red points represent the stations where vertical profile samples were collected. The yellow oval circles indicated the gyre areas (surface chlorophyll concentration ≤0.07 mg m^−3^).
(TIF)

Figure S3 **Side scatter versus green fluorescence obtained for a natural viral sample (A), 0.02 µm filtered TE buffer (B), a pure-cultured marine roseophage RDJL Phi 1 lytically infecting** ***Roseobacter denitrificans*** **OCh114 (C), a natural heterotrophic prokaryotic sample (D), and red fluorescence versus green fluorescence obtained for the natural heterotrophic prokaryotic sample (E); all were stained with SYBR- Green I.** Side scatter versus red fluorescence (F) and orange fluorescence versus red fluorescence (G) obtained for a natural autotrophic picoplankton sample. One micron fluorescent beads were added as an internal reference. Abbreviations: HFV, high fluorescence viruses; LFV, low fluorescence viruses; HetProk, heterotrophic prokaryotes; AutoProk, autotrophic prokaryotes; Proc, *Prochlorococcus*; Syn, *Synechococcus*, Euk, picoeukaryotes.
(TIF)

Table S1 The parameters and their numbers for the DISTLM forward analysis in the Table 2 & 3.
(DOCX)
