# Supplementary figures and images for: Horizontal and Vertical Distribution of Marine Virioplankton: A Basin Scale Investigation Based on a Global Cruise
Source: PLoS One. 2014 Nov 3;9(11):e111634. doi: 10.1371/journal.pone.0111634 (PMC4218788; doi:10.1371/journal.pone.0111634)

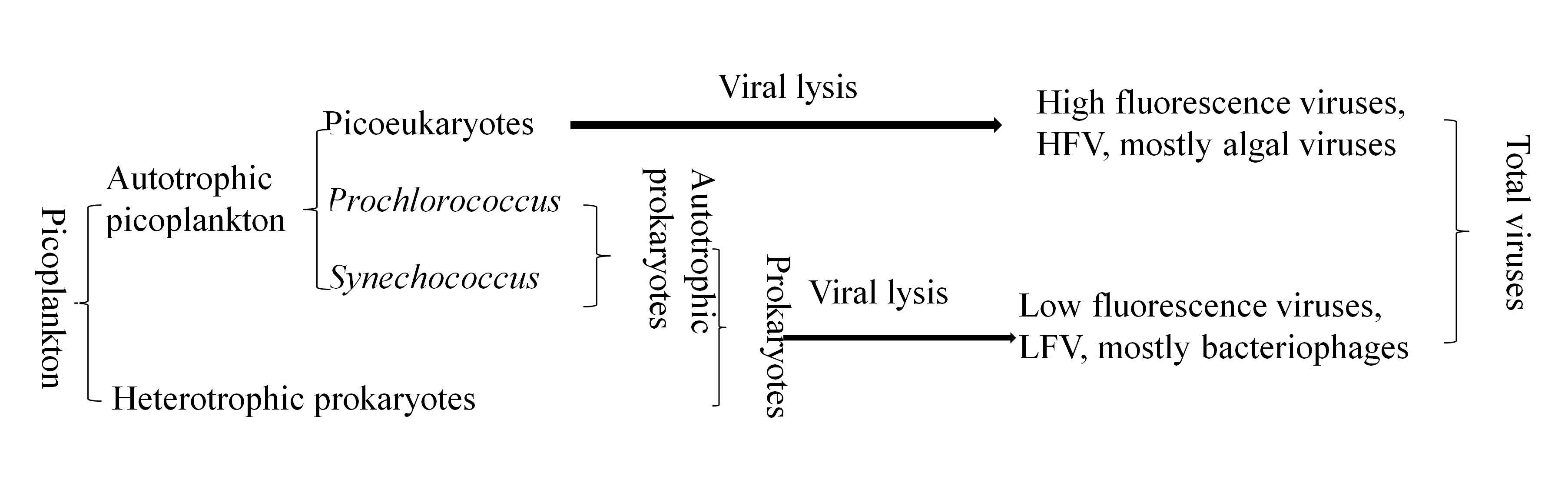

Supplement: Figure S1 — The hierarchical tree of how the samples fractionated. (TIF) [file pone.0111634.s001.tif]

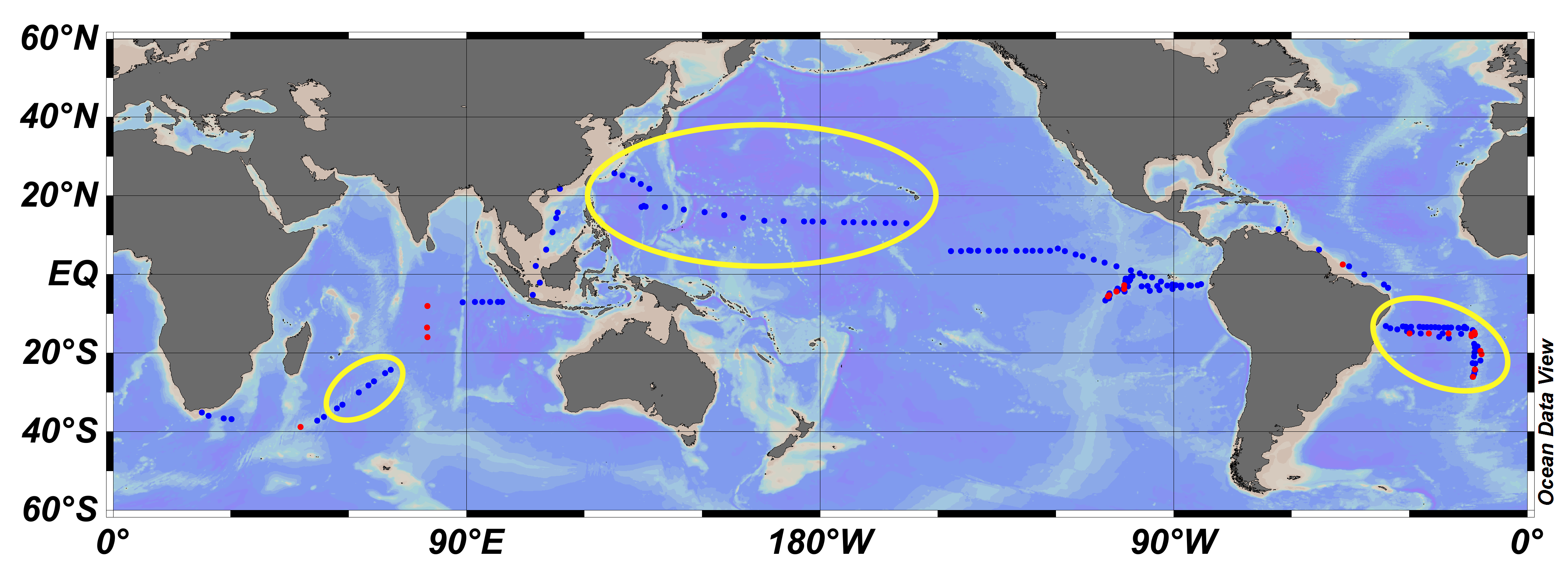

Supplement: Figure S2 — Sampling stations of the global cruise. The blue points represent the stations where surface samples were collected. The red points represent the stations where vertical profile samples were collected. The yellow oval circles indicated the gyre areas (surface chlorophyll concentration ≤0.07 mg m−3). (TIF) [file pone.0111634.s002.tif]

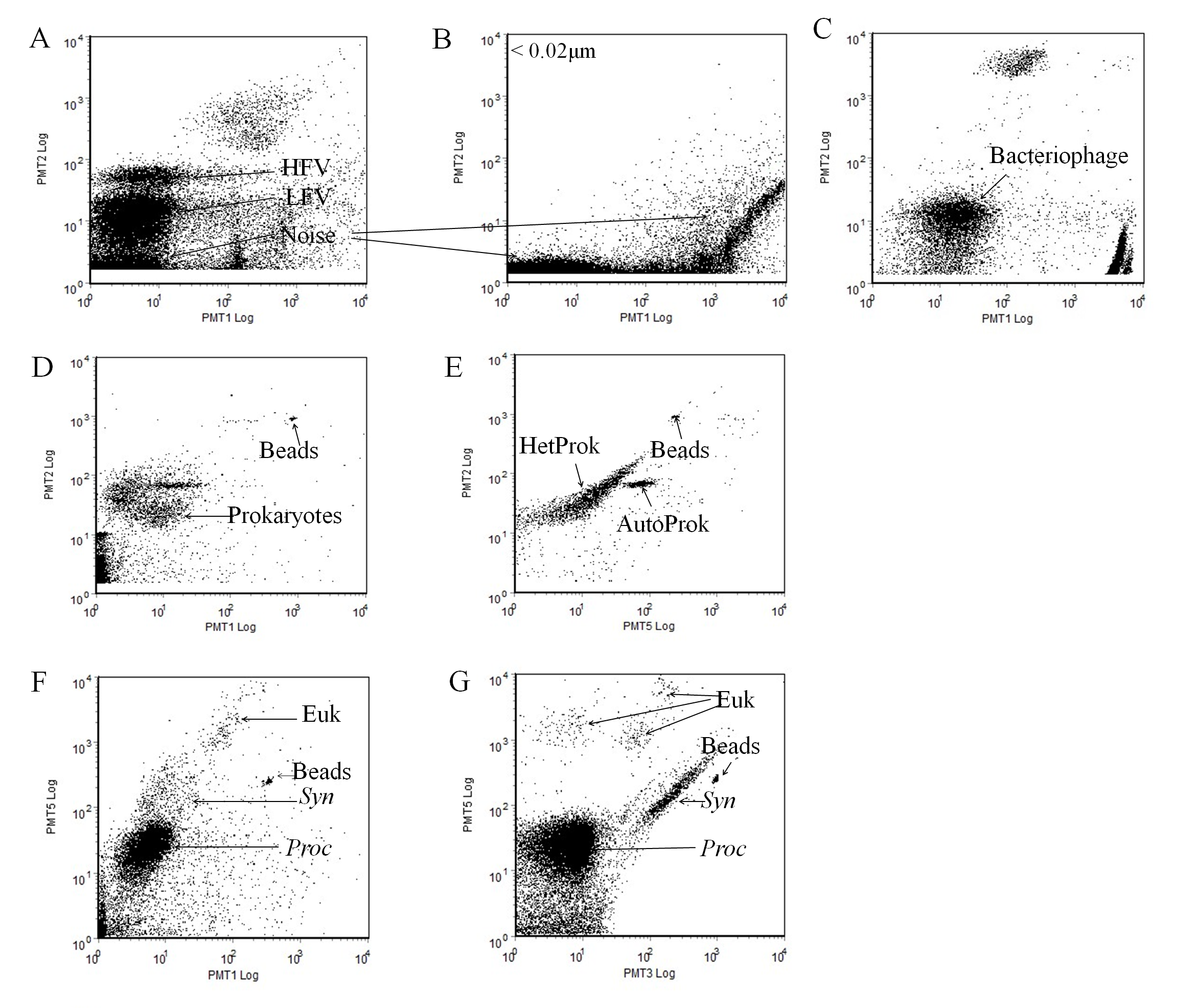

Supplement: Figure S3 — Side scatter versus green fluorescence obtained for a natural viral sample (A), 0.02 µm filtered TE buffer (B), a pure-cultured marine roseophage RDJL Phi 1 lytically infecting Roseobacter denitrificans OCh114 (C), a natural heterotrophic prokaryotic sample (D), and red fluorescence versus green fluorescence obtained for the natural heterotrophic prokaryotic sample (E); all were stained with SYBR- Green I. Side scatter versus red fluorescence (F) and orange fluorescence versus red fluorescence (G) obtained for a natural autotrophic picoplankton sample. One micron fluorescent beads were added as an internal reference. Abbreviations: HFV, high fluorescence viruses; LFV, low fluorescence viruses; HetProk, heterotrophic prokaryotes; AutoProk, autotrophic prokaryotes; Proc, Prochlorococcus; Syn, Synechococcus, Euk, picoeukaryotes. (TIF) [file pone.0111634.s003.tif]
