# Supplementary material for: Horizontal and Vertical Distribution of Marine Virioplankton: A Basin Scale Investigation Based on a Global Cruise
Source: PLoS One. 2014 Nov 3;9(11):e111634. doi: 10.1371/journal.pone.0111634 (PMC4218788; doi:10.1371/journal.pone.0111634)
Supplement: Table S1 — The parameters and their numbers for the DISTLM forward analysis in the Table 2 & 3. (DOCX) [file pone.0111634.s004.docx]

Table S1

|  | Regions | Parameters | Number |
| --- | --- | --- | --- |
| Surface ocean | Global ocean | *Prochlorococcus*, *Synechococcus*, picoeukaryotes and heterotrophic prokaryotes | 205 |
|  | Coastal/shelf/  upwelling | *Prochlorococcus*, *Synechococcus*, picoeukaryotes and heterotrophic prokaryotes | 115 |
|  | Gyrs | *Prochlorococcus*, *Synechococcus*, picoeukaryotes and heterotrophic prokaryotes | 90 |
| Water column | Global ocean | *Prochlorococcus*, *Synechococcus*, picoeukaryotes, heterotrophic prokaryotes and depth | 252 |
|  | Epipelagic | *Prochlorococcus*, *Synechococcus*, picoeukaryotes, heterotrophic prokaryotes and depth | 152 |
|  | Mesopelagic | Heterotrophic prokaryotes and depth | 56 |
|  | Bathypelagic | Heterotrophic prokaryotes and depth | 44 |
|  | Coastal/shelf/upwelling | *Prochlorococcus*, *Synechococcus*, picoeukaryotes, heterotrophic prokaryotes and depth | 120 |
|  | Epipelagic | *Prochlorococcus*, *Synechococcus*, picoeukaryotes, heterotrophic prokaryotes and depth | 74 |
|  | Mesopelagic | Heterotrophic prokaryotes and depth | 28 |
|  | Bathypelagic | Heterotrophic prokaryotes and depth | 18 |
|  | Gyres | *Prochlorococcus*, *Synechococcus*, picoeukaryotes, heterotrophic prokaryotes and depth | 132 |
|  | Epipelagic | *Prochlorococcus*, *Synechococcus*, picoeukaryotes, heterotrophic prokaryotes and depth | 78 |
|  | Mesopelagic | Heterotrophic prokaryotes and depth | 28 |
|  | Bathypelagic | Heterotrophic prokaryotes and depth | 26 |
